# Nutritional Assessment and Support in Children with Chronic Kidney Disease: The Benefits of Working with a Registered Dietitian

**DOI:** 10.3390/nu15030528

**Published:** 2023-01-19

**Authors:** Marta Suárez-González, Flor Ángel Ordoñez-Álvarez, Helena Gil-Peña, Sara Carnicero-Ramos, Lucía Hernández-Peláez, Sonia García-Fernández, Fernando Santos-Rodríguez

**Affiliations:** 1 Department of Pediatrics, Hospital Universitario Central de Asturias, 33011 Oviedo, Spain; 2 Pediatric Research, Instituto de Investigación Sanitaria del Principado de Asturias, 33011 Oviedo, Spain; 3 Medicine Área, Universidad de Oviedo, 33006 Oviedo, Spain

**Keywords:** dietitian, nutritional support, chronic kidney disease, pediatrics, renal diet

## Abstract

Background: An unbalanced dietary pattern, characterized by high animal protein content: may worsen metabolic control, accelerate renal deterioration and consequently aggravate the stage of the chronic kidney disease (CKD) in pediatric patients with this condition. Aim: to assess the effect of a registered dietitian (RD) intervention on the CKD children’s eating habits. Methods: Anthropometric and dietetic parameters, obtained at baseline and 12 months after implementing healthy eating and nutrition education sessions, were compared in 16 patients (50% girls) of 8.1 (1–15) years. On each occasion, anthropometry, 3-day food records and a food consumption frequency questionnaire were carried out. The corresponding relative intake of macro- and micronutrients was contrasted with the current advice by the European Food Safety Authority (EFSA) and with consumption data obtained using the Spanish dietary guidelines. Student’s paired t-test, Wilcoxon test and Mc Nemar test were used. Results: At Baseline 6% were overweight, 69% were of normal weight and 25% were underweight. Their diets were imbalanced in macronutrient composition. Following nutritional education and dietary intervention 63%, 75% and 56% met the Dietary Reference Values requirements for fats, carbohydrates and fiber, respectively, but not significantly. CKD children decreased protein intake (*p* < 0.001), increased dietary fiber intake at the expense of plant-based foods consumption (*p* < 0.001) and a corresponding reduction in meat, dairy and processed food intake was noticed. There were no changes in the medical treatment followed or in the progression of the stages. Conclusions: RD-led nutrition intervention focused on good dieting is a compelling helpful therapeutic tool to improve diet quality in pediatric CKD patients.

## 1. Introduction

Chronic kidney disease (CKD), characterized as irregularities of kidney construction or capability (sediment, image, histology), present for at least three months, is arranged in light of cause, glomerular filtration rate (GFR) class and albuminuria class (CGA) [1] (Table 1 and Table 2).

Regular assessment of nutritional status and provision of suitable nutrition are key parts in the overall management of children with CKD. Nutritional management for children suffering from this pathology should be aimed at preventing protein-energy malnutrition and meeting the patient’s micronutrients needs. Overnutrition, described as excess weight and the long-term implications of an undesirable eating routine and way of life increasingly worrying to the pediatric CKD populace. Consideration of this subject should be integrated into the nutrition management plan [2].

Management objective in youngsters with CKD include slowing disease progression, anticipation and therapy of complications, optimizing growth, development and quality of life. Providing a balanced diet through nourishing support [3], is basically critical to accomplish these objectives [4].

Furthermore, the biggest contributor to the progressive decline in GFR is the high intake of animal proteins [5]. Thus, the treatment to forestall or address metabolic acidosis involves dietary handling by reducing or eliminating the consumption of animal proteins and increasing the consumption of plant-based proteins [6,7]. Moreover, the dietary limitations of phosphorus and protein are shown to reduce the deterioration of kidney capability and to likely bring down the risk of end-stage renal disease in patients with CKD [8].

The three sources of dietetic phosphorus are organic phosphorus from animal protein (bioavailability 40–60%), organic phosphorus from plant-based foods (bioavailability 20–40%) and inorganic phosphorus found in additives and processed foods (bioavailability ≈ 100%). The reason why the bioavailability in plant-based foods is the lowest of the three sources is because humans do not produce phytase, which is the enzyme that degrades phytates in plant food [9]. Inorganic phosphorus, added to processed foods, is almost entirely absorbed and may add up to 1000 mg/d of phosphorus from additives alone [10]. Choosing foods that contain phosphorous in lower bioavailability and without phosphate additives is highly recommended [11].

Nutritional habits that are more plant-based, with a higher content of grains and fibers and lower in meat (including processed meat), sodium and refined sugar, are currently recommended according to different clinical guidelines to prevent chronic disease [12,13].

The role of the dietitian is to assess an adequate nutritional status and growth, provide referral-based education for dietary requirements or potentially limitations, assess adherence to these proposals, and teach and counsel children, their families and doctors [14,15,16,17].

Evidence from studies using dietetic intervention reveal that regular nutrition support brings about adherence and improved outcomes in the general pediatric population [18,19,20,21]. Nonetheless, there are limited investigations of CKD children, even though a nutrition therapy is highly recommended for them [22,23].

Furthermore, it has been shown that if the dietitian provides the individualized dietary evaluation and counseling in CKD patients the intervention is cost-effective [24,25,26,27,28,29], improves control of diabetes and hypertension, slows CKD progression and delays the need of kidney replacement therapy in later stages [30,31,32,33,34,35,36,37].

The goal of our review was to explore the changes in the eating habits, diet and nutritional status of pediatric patients with CKD after executing a nutrition education program carried out by an accomplished enrolled dietitian, focusing on achieving a balanced nutritious diet that gives sufficient amounts of nutrients. We hypothesized that this intervention would likewise assist pediatric patients with CKD to meet the Spanish dietary recommendations for children.

## 2. Patients and Methods

### 2.1. Participants

The review was implemented in the Pediatric Nephrology and Clinical Nutrition Unit of at the Central University Hospital of Asturias (Spain), between July 2017 and December 2020.

Requested inclusion criteria were: males and females with ages ranging from 1 to 18 years with CKD, under the care of the specialist pediatric nephrology clinic.

The exclusion criteria were: subjects with a different condition from CDK, who rejected the clinical follow-up and any who had acquired past dietary guiding from the RD at the unit.

The Clinical Research Ethics Committee of the Principality of Asturias authorized the study (CEImPA 2022.197).

### 2.2. Nutritional Assessment

Anthropometric data were gathered along with a dietary evaluation. Calibrated scales were utilized to quantify weight and height data with subjects wearing only underwear and no footwear. From the information, body mass index [BMI = weight/height^2^ (m)], its Z-score (Z-score = mean data−median of reference (P50)/SD) and height Z-score according with World Health Organization (WHO) and Spanish growth studies (Carrascosa et al. 2010 [38] and Carrascosa et al. 2017 [39]), were calculated using the pediatric nutritional application of the Spanish Society of Gastroenterology and Pediatric Nutrition (SEGHNP) website [40]. Subjects were classified by SD BMI Z-score as malnourished (≤−1 SD), normal nutritional status (−0.99 to 0.99 SD), overweight (1–1.99 SD) and obese (≥2 SD).

Dietary evaluation was undertaken using a 3-day dietary study and food consumption frequency questionnaire. The 3-day dietary study recorded all foods eaten by the subjects over a 3-day period, including one day of the weekend. Subjects weighed foods using a scale accurate to 1 g and also used estimated household data supported with a list of typical portion sizes of common food items. Records were analyzed using a Spanish dietary analysis program (ODIMET) [41] calculating the relative contributions of macro and micronutrients. The findings were compared with dietary reference values (DRVs) for nutrient intake established by the European Food Safety Authority (EFSA) [42]. The consumption information obtained from the food frequency questionnaire were contrasted with the Spanish dietary guidelines [43]. This survey, though not validated, was completed in the consultation by the RD who asked the patients and their family members how often they consumed different types of food (fruit, vegetables, nuts, legumes, rice, pasta, potato, bread, meat, fish, eggs, dairy products) and processed food (fast food, snacks and soft drinks, pastries, biscuits, chocolate and breakfast cereals).

### 2.3. Nutritional Education

Patients got oral and written dietary nutrition information and guidance about eating a healthy and balanced diet through individualized food instruction based on the Healthy Eating Plate recommendations by the Harvard School of Public Health [44]. Additionally, the importance of strict adherence to the treatment was explained. Written instances of age-specific sample diets were given as well. The dietary session lasted 1 h and the recommendations were directed individually based on their eating habits.

At six months from the first intervention, the patients and their families got back to the pediatric nutrition clinic. In this second meeting further advice was given to inspire and build up behavioral habits and to support recently implemented changes in their eating habits. Likewise, they were given a further 3-day dietary study to be completed before the last review session, which was scheduled six months later. The total follow-up time was one year. Complete nutritional assessment was performed before the intervention and the following year. The data were collected in a prospective manner.

### 2.4. Statistics

Statistical analysis was performed utilizing R, version 3.6.0 (R Foundation for Statistical Computing, Vienna, Austria) [45]. A descriptive analysis of the relative and absolute frequency distributions for qualitative variables and position and dispersion measures of the quantitative variables was performed. Paired Student’s t-test and Wilcoxon test for quantitative variables and McNemar test for qualitative variables were applied to determinate the distribution homogeneity between qualitative variables with more than two levels and for paired samples. A *p* < 0.05 was considered statistically significant.

## 3. Results

### 3.1. Patients

Firstly, a total of 22 subjects diagnosed with CKD were recruited [9 (41%) girls and 13 (59%) boys]. After the first intervention, 6 declined to continue. The final cohort included 16 subjects, 50% of whom were girls. The median age of patients at the start of the study was 8.4 years (range, 1–15 years) and the median age at diagnosis of CKD was 1.5 years (range, 1–10), caused by congenital anomalies of kidney and urinary tract in 37% of the cases. Other etiologies were bilateral renal cortical necrosis (18%), in two cases due to septic shock, and hemolytic uremic syndrome with diarrhea, cystinuria, enamel-renal syndrome, congenital heart anomaly or autosomal recessive polycystic kidney disease in one patient each. At the beginning of the study, 7 patients were at stage 2 of CKD, 8 at 3 stage and 1 CAKUT case at stage 4.

Regarding nutritional state at baseline, according to WHO standards, 6% were overweight, 69% presented normal weight and 25% were malnourished. Toward the end of the review, 13% were defined as overweight, 50% well-nourished and 37% malnourished, but BMI values between baseline [mean (SD) BMI Z-score −0.55 (1.10)] and after the year of intervention [mean (SD) BMI Z-score −0.52 (1.46)] were not significantly different. There were also no significant differences according to the other reference values used, [mean (SD) BMI Z-score 0.21 (0.45)] by Carrascosa et al. 2010 38 and [mean (SD) BMI Z-score 0.23 (0.76)] by Carrascosa et al. 2017 39.

### 3.2. Dietary Intake

#### 3.2.1. Nutrients

According to DRVs, baseline diets were imbalanced in macronutrient composition for fat and carbohydrates (expressed in percentage of total daily calorie intake), proteins (expressed in g/kg body weight/day) and fiber (expressed in g/day). All participants exceeded recommended protein intakes, 68% exceeded advice fat intakes, 31% did not cover the minimum recommendations of carbohydrates and 63% did not intake an adequate amount of fiber. After the nutrition intervention, the compliance with the recommendations increased from 31% to 63% of patients for fat, from 69% to 75% for carbohydrates and from 38% to 56% for fiber, but not significantly.

**Figure 1:**
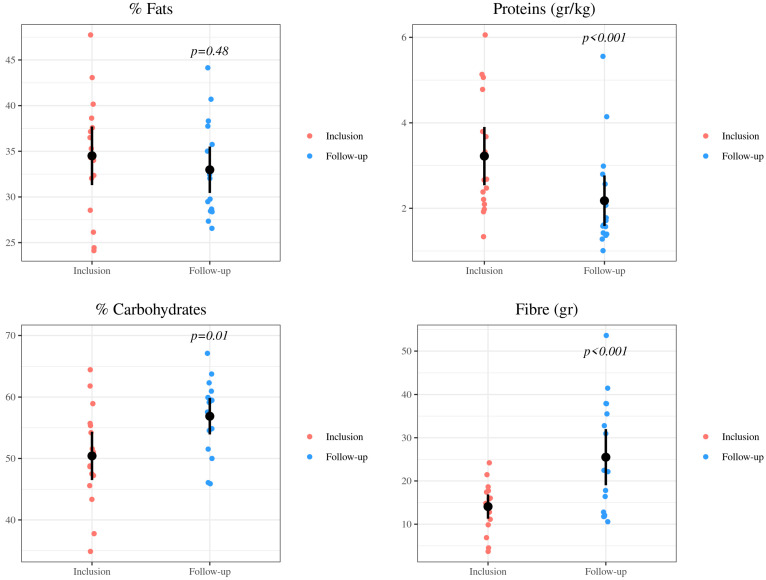
Ingestion of macronutrients at baseline and last follow-up after the nutrition intervention. Fats (% fats), carbs (% carbohydrates) and grams of protein per kilogram of body weight (protein/kg), as well as grams of fiber each day (fiber), consumed by 16 pediatric subjects with CKD.

All children exceeded the protein’s recommendations, both before and after the intervention, but a significant decrease was observed [mean (SD) −1.04 (0.93) g/kg/day, *p* < 0.001]. Mean (SD) protein intake at baseline was 3.22 (1.39) g/kg/day while at the end of the study it was 2.18 (1.21) g/kg/day (Figure 1).

Analyzing the proportion of nutrients with respect to the total caloric value of the diet, an insignificant decrease of 4% in total amount of fat was observed, but contribution of saturated fat decreased by 25%. Carbohydrate intake increased by 13% and fiber intake by 45% after undergoing food education (Figure 1). The results of the energy, macro and micronutrients studied are provided in Table 3.

Regarding minerals, compliance with calcium, phosphorus, potassium and sodium were 44%, 100%, 69% and 81%, respectively. These values remained practically constant at the end of the study, and no statistical significance was found. An increase in the consumption of potassium [mean (SD) 579.28 (755.92) mg/day, *p* = 0.01] was observed after one year (Table 3).

#### 3.2.2. Foods

After comparing consumption habits before and after the intervention, a general increase in plant-based varieties and a decrease in the eating of animal source foods was observed (Table 4).

**Figure 2:**
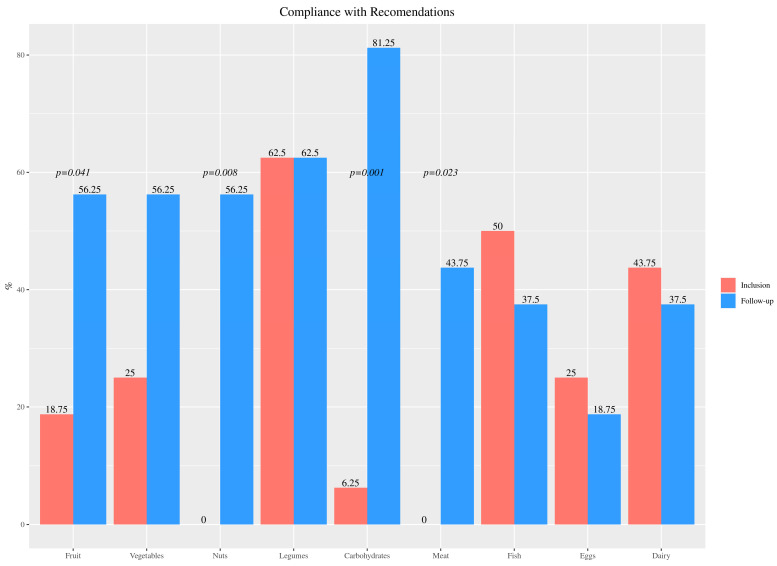
Percentage of participants with chronic kidney disease meeting the Spanish nutritional recommendations for fruit, vegetables, nuts, legumes, complex carbs (grains and tubers), meat, fish, eggs, and dairy, at baseline and last follow-up after nutrition intervention.

At baseline, most children (78%) did not meet Spanish nutrition recommendations in terms of fruit and vegetable consumption and none of the participants consumed nuts. By contrast, 1 year after the nutritional education, 56% complied with the recommendations for these foods (fruit and vegetables *p* = 0.041; nuts *p* = 0.008). Findings indicated that all participants ingested excessive amounts of meat and 56% also consumed dairy products in excess of recommendations. In this case, 50% of the subjects consumed ≥3 portions of fish per week at baseline, with a slight decrease to 38% meeting fish intake recommendations for healthy children. The intake of meat decreased (*p* = 0.023) and that of starches (comprising of cereals and tubers) increased by the one-year follow-up, with 81% of children meeting the recommendations for complex carbohydrates (*p* = 0.001) (Figure 2).

Other dietary data showed that 31% and 81% of children consumed chocolate drinks and sugared dairy desserts daily, respectively, all of them included biscuits in their breakfasts two to seven times a week, and 69% included pastries and 31% sugary cereal breakfast with the same frequency. Concerning the consumption of sugar sweetened beverages, 50% consumed bottled juices and soft drinks at least once a week. During the weekend also, 44% of children consumed fast food, 50% savory snacks and 69% chocolates.

**Figure 3:**
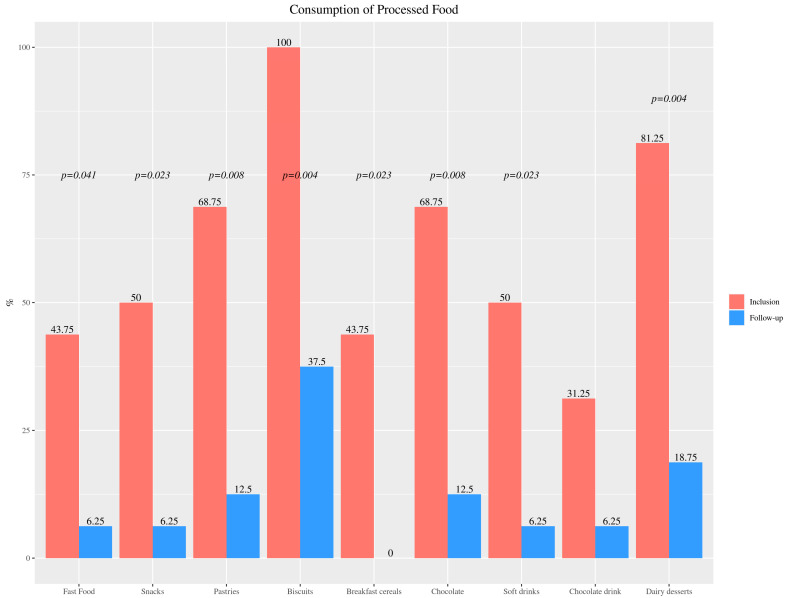
Percentage of the 16 participants with chronic kidney disease that consumed different processed foods (fast food, snacks and soft drinks at least once a week; pastries, biscuits, chocolate and breakfast cereals 2 to 7 times a week; chocolate drinks and dairy desserts daily) at baseline and last follow-up.

Following nutrition education, significant changes in eating patterns were noticed, with a diminishing in the consumption of all highly processed manufactured products. Figure 3 shows changes in the intake of commonly consumed processed foods at baseline and last follow-up.

## 4. Discussion

Clinical nutrition therapy is essential for CKD children because it might slow the progression of the disease through cautious observing of protein, calcium, phosphorus, potassium and sodium [46]. The fundamental contribution in our review is to give information to physicians about the current poor diet that children with CKD follow and that it is necessary to change this tendency working together with an RD. This is a crucial aspect in the intervention for these patients because nutrition aids in the delay of the disease’s progression, improves a patient’s wellbeing, decreases the risk of complications and prevents comorbidities and mortality [47].

Specifically, the nutritional education provided by the RD in our study managed to make the children with CKD to improve the quality of their diet, reducing proteins consumption to the detriment of animal source foods and increasing carbohydrates and fiber intake at the expense of plant-based food. Therefore, the most relevant aspect in our study is to demonstrate that it is possible to achieve a change in the patients eating habits in order to improve the progression of their disease.

On the other hand, no change was achieved in their nutritional status. Nutritional status in children with CKD might also be impacted by several factors, including loss of hunger, altered gastrointestinal motility, malabsorption, intestinal dysbiosis, and uremic irregularities of energy, protein, lipid, and starch metabolism. Evaluation of nutritional status in children with CKD is complex [2,48], and should ideally be conducted jointly by a pediatric nephrologist and a pediatric dietitian experienced in the nutritional management of this type of patient [49].

Analyzing the diet reported by the patients at the beginning of the study, we observed an unbalanced dietary pattern characterized by being rich in proteins and saturated fats and deficient in complex carbohydrates and fiber. CKD children with protein intake exceeding recommended levels were also observed in the literature [50].

All children also exceed the age-specific average daily phosphorous intake level resulting from their high protein consumption. Foods rich in protein contain a high phosphorus load, and protein restriction is usually not recommended for children with CKD for promotion of growth, but on the other hand, excessive dietetic protein consumption might contribute to CKD progression and impact the control of metabolic bone disease [51].

Furthermore, the popularity of very-high-protein diets warrants reviewing their potential detrimental effect on patients who use them. These eating regimens differ broadly and may contain excessive amounts of protein, phosphorus, or potassium. There is no published evidence of the long-term metabolic impact of them. However, they have been found to deliver a marked acid load to the kidney, increase the risk of stone formation, advance negative calcium balance, increment bone loss and GFR [52].

Generally, guidelines and recommendations agree that controlling protein consumption ought to be an objective in the dietetic management of CKD. This perspective depends on the speculation that decrease protein consumption slows progression of kidney disease. Indeed, even a 0.1 to 0.2 g/kg/day decrease in dietary protein from baseline seems to bring about huge metabolic improvement and longer conservation of kidney wellbeing [53]. We achieved a 1.04 g/kg/day reduction in protein consumption in children with CKD after the nutrition intervention. Due to this, a decrease of 110 mg of daily phosphorus was also achieved.

Furthermore, in a systematic review and meta-analysis of control trials, a low-protein dietary pattern seems to enhance the conservative management of non-dialysis-dependent CKD and might be considered as a potential choice for CKD patients who wish to stay away from or defer dialysis initiation and to slow down the progression of CKD, while the risk of protein-energy wasting and cachexia remains minimal [54].

On the other hand, in addition to a reduction in the protein consumption in the diet of our patients, a decrease in saturated fat and increase in carbohydrates and fiber was also achieved. These changes made the diet more balanced.

Regarding the other minerals studied, without considering the sodium from added salt during food cooking, 19% of the children in our study exceeded the recommended maximum sodium intake due to the salt naturally contained in food and with that added in manufactured products. The global consumption of salt would be much higher if we had taken into account the added salt. Thus, our results show that a greater effort is required to reduce dietary sodium consumption by children. Behavioral strategies such as avoiding processed food and reading food labels should be encouraged. Importantly, one achievement was that the change in habits resulted in 94% of the children compiling with the recommended maximum daily intake of sodium by the end of the study.

Although restriction of potassium consumption is also recommended for children with CKD who are at risk of hyperkalemia [2], the true benefit of this is not clear, taking into consideration that a diet with a high content of potassium-rich foods, such as plant-based low-protein diets, can be as beneficial on the prognosis [55,56].

Vegetables and fruits are the main sources of dietary potassium [57], and instead of restricting them, we recommend pre-soaking the vegetables for a period of 12 and 24 h, with at least one change of water, double cooking with plenty of water and cutting them into small pieces and discarding the cooking broth to allow them to reduce their potassium content to acceptable levels, which would allow their inclusion in the diet of patients with CKD. These recommendations are based on the loss of potassium and other soluble minerals in food due to passing it through cooking water [58]. For this reason, although a slight increase in potassium has been in the dietary analysis, due to increase in fruit and vegetable consumption, it does not reflect the actual dietary potassium consumed by our patient.

In terms of foods, most children included in our study did not achieve Spanish recommendations for the fruit, vegetables, nuts and cereals intake but exceeded recommended intake for meat and processed products. This seems to be a general characteristic of the Spanish population lately, as exhibited by the recent ANIBES study [59].

Recommending an eating style based on the Healthy Eating Plate can help patients make positive changes. This dietary pattern is supported by strong evidence that promotes the intake of unprocessed foods, fruits and vegetables, plant-based fats and proteins, whole grains, legumes and nuts. Added sugars ought to be restricted to under 5% to 10% of daily caloric intake. Including monounsaturated fats, such as olive oil, avocados and nuts and omega-3 unsaturated fats, loke flax, oily fish and nuts consumption should be highlighted. An emphasis on foods rather than macronutrients can assist patients in understanding a healthy diet [44].

Taking on and sticking to another eating pattern requires the capacity to persuade patients to make changes that will work on their wellbeing and forestall morbidities, although the changes might be uncomfortable for the patient. Adequate counseling about the rationale of the recommendations and how the patient will benefit are fundamental to convey. Similarly, equally as significant is to evaluate the patient’s maintenance and comprehension of the dietetic advice. Giving alternative food options tailored to the patient’s preferences to replace restricted foods is more useful than focusing on the limitations. Giving replacement instructions to the patient is basic to attain and maintain patient compliance and achieve successful dietary management [47].

The traditional dietary management of the patients with CKD focuses on providing the quantity of energy and protein within the diet, and the restriction of single micronutrients, with little mention of dietary quality. Thus, the dietitian has a more significant function than merely to show the recommended amount of nutrients, but is also essential to translate this message into food.

The current review showed that nutritional education brought about huge upgrades in these patients eating patterns and ways of life. Positive changes included a significant decrease in the processed foods intake, specifically fast food, snacks, soft drinks, pastries, biscuits, breakfast cereals and chocolate drinks. Reductions in the consumption of dairy, at the expense of milk, cheese, sweetened yogurts and sweetened dairy desserts, and meats (red and white) and processed meat and derivatives (sausages, cold cuts, sausages and hamburgers), were likewise demonstrated. Specifically, the patients went from consuming meat daily to three times a week, and in terms of dairy products, a reduction of one and a half portions per day was achieved. This was relevant because the content of phosphorus is high in dairy products (about 100 mg of phosphorus is found in 100 mL of milk and >500 mg per 100 g of cheese) [60].

Consequently, due to reduced consumption of animal source foods, the current study also showed an increased consumption of plant-based food varieties including fruits, vegetables, nuts, rice, pasta and bread. The consumption of these good food varieties in the review populace at baseline had been extremely low, even missing. The nutritional education carried out in our study by the RD was able to make the children with CKD double the consumption of cereals and tubers portions, as well as increase one more portion of fruit, another of vegetables per day and almost three more servings of nuts a week.

This is the main RD started study assessing the efficacy of nutritional education among Spanish children diagnosed with CKD. There are no other equivalent examinations by which its efficacy can be assessed. All subjects were seen by the same RD, with uniform nutritional counseling in all cases.

The main limitations of the current review were that the sample size was small and there was no control group. Likewise, interest predisposition is a possibility because parenteral inspiration, financial status, accessibility to fresh products and cooking skill may have impacted on the willingness to take part. We also consider a follow-up bias in a period of one year. More studies would be necessary to assess whether the nutritional intervention is related to changes in long-term renal function and in the nutritional status (better Z score for height and decrease the percentage of underweight and obesity).

Suitable dietary counseling for CKD patients and their families should focus on a healthy diet by deciding on quality natural food, which provide adequate nutrition. Our observations, along with the review of the literature, highlights the need for CKD patients to receive personalized nutrition advice, comprising of a fundamental tool for teaching the patient to increase the consumption of fruit, vegetables and cereals to achieve a greater adherence to the recommendations of the healthy eating pattern.

## 5. Conclusions

This study has demonstrated that nutrition counseling provided by a RD helped CKD children to adopt healthier eating habits. Appropriate nutrition education for these patients should focus on the global intake of their diet, such that, in addition to their being healthy, it is nutritionally adequate and individualized according to results of the nutritional assessment.

Our analysis showed that pediatric patients with CKD consumed more dietetic animal protein and fats from meat and dairy sources than recommended by national and European guidelines. This together with the fact that the consumption of natural plant-based foods is much lower than the recommendations, emphasizes the need for efficacious nutritional intervention strategies in this patient population.

## Figures and Tables

**Table 1:** CKD criteria (present for more than 3 months).

Kidney damage markers	Albuminuria (ACR > 30 mg/g) Abnormal urine sediment (e.g., hematuria, red cell casts, etc.) Electrolyte and different anomalies because of tubular issues Irregularities recognized by histology Structural abnormalities distinguished by imaging History of kidney transplantation
Decreased GFR	GFR < 60 mL/min/1.73 m^2^ (GFR categories G3a-G5)

**Table 2:** GFR categories in CKD.

GFR Category	GFR (mL/min/1.73 m^2^)	Terms
G1	>90	Normal or high
G2	60–89	Mildly diminished
G3a	45–59	Mildly to moderately diminished
G3b	30–44	Moderately to severely diminished
G4	15–29	Severely diminished
G5	<15	Kidney failure

Abbreviations: CKD, chronic kidney disease; GFR, glomerular filtration rate; ARC, albumin to creatinine ratio. The G1 and G2 categories refer to other signs of kidney damage. Categories G3a to G5 are included as CKD criteria [1].

**Table 3:** Daily energy and nutrients consumption at baseline and last follow-up after nutrition intervention.

Energy and Nutrients		Inclusion	Follow-Up	*p*-Value
Energy	Energy (kcal)	1683.64 (511.89)	1783.22 (694.03)	0.4
Proteins	Proteins (g)	67.34 (20.45)	53.21 (19.77)	0.01
	Proteins/kg (g)	3.22 (1.39)	2.18 (1.21)	<0.001
	Proteins (%)	16.29 (1.91)	12.48 (2.30)	<0.001
Fats	Fats (g)	64.20 (23.03)	64.29 (27.38)	0.99
	Fats (%)	34.51 (6.55)	32.97 (5.19)	0.48
	Saturated fat (g)	20.53 (8.32)	16.02 (7.96)	0.14
	Saturated fat (%)	11.03 (2.81)	8.29 (3.38)	0.05
	MUFAs (g)	25.23 (11.56)	30.50 (14.34)	0.14
	MUFAs (%)	13.54 (4.67)	16.28 (3.27)	0.11
	PUFAs (g)	7.31 (4.18)	11.74 (13.44)	0.18
	PUFAs (%)	4.04 (2.33)	4.66 (1.94)	0.44
Carbohydrates	Carbohydrates (g)	209.19 (66.81)	247.93 (102.21)	0.03
	Carbohydrates (%)	50.42 (8.03)	56.88 (6.06)	0.01
	Fiber (g)	14.08 (5.73)	25.50 (13.26)	<0.001
Minerals	Sodium (mg)	963.81 (464.00)	861.19 (502.50)	0.37
	Potassium (mg)	2215.91 (884.50)	2795.19 (958.63)	0.01
	Calcium (mg)	734.42 (279.28)	578.40 (235.18)	0.07
	Phosphorus (mg)	961.33 (670.36)	851.08 (283.28)	0.82

Data of the 16 CKD pediatric patients, are presented as: mean (SD). Monounsaturated fatty acids (MUFAs), Poly-Unsaturated Fatty Acids (PUFAs).

**Table 4:** Frequency of consumption frequency of type of food at the beginning and last follow up after nutrition intervention.

Food Group	Foods	Inclusion	Follow-Up	*p*-Value
Fruit and vegetables	Fruit/day	1.59 (1.29)	2.72 (0.84)	<0.001
	Vegetable/day	0.78 (0.86)	1.72 (0.36)	<0.001
Plan protein sources	Legume/week	3.28 (1.37)	3.03 (1.02)	0.68
	Nuts/week	0.19 (0.40)	2.72 (2.62)	0.01
Animal protein sources	Meat/week	6.75 (0.68)	3.29 (2.08)	<0.001
	Fish/week	2.56 (1.12)	2.5 (0.98)	0.79
	Eggs/week	2.44 (1.76)	2.16 (1.01)	0.51
	Dairy/day	3.66 (1.43)	2.09 (0.92)	<0.001
Cereal and tubers	Rice/week	1.16 (0.68)	2.38 (1.27)	0.01
	Pasta/week	1.16 (0.77)	2.84 (1.27)	<0.001
	Potato/week	4.84 (2.10)	5.31 (1.67)	0.47
	Bread/day	1.13 (0.96)	2.75 (1.34)	<0.001
	Total carbohydrates/day	2.47 (1.49)	4.28 (1.38)	0.01

Data of the 16 children with CKD are presented as: mean (SD).
